# Drug-Related Death Bereavement: A Commentary by Kelly Thomas on Titlestad, Stroebe, and Dyregrov’s Article: How Do Drug-Death-Bereaved Parents Adjust to Life without the Deceased? A Qualitative Study

**DOI:** 10.1177/0030222820946550

**Published:** 2020-08-09

**Authors:** J. Kelly Thomas, Kristine B. Titlestad, Margaret Stroebe, Kari Dyregrov

**Affiliations:** 1 San Antonio, Texas, United States; 2 Faculty of Health and Social Sciences, Western Norway University of Applied Sciences, Bergen, Norway; 3 Department of Clinical Psychology and Experimental Psychopathology, University of Groningen, Groningen, the Netherlands; 4 Faculty of Health and Social Sciences, Western Norway University of Applied Sciences, Bergen, Norway

## Introduction by the Authors

Kelly Thomas first contacted us briefly—but to us so poignantly—about an earlier publication of ours on Drug-Related Death (DRD) bereavement entitled *Sounds of silence. The ‘special grief’ of drug-death bereaved parents* (Titlestad et al., 2020). He commented:

It is coming up on the 3 years since we lost our daughter at age 21 in a DRD. I have found my wife listening to the song your paper refers to more times than I would like to count. I cried as I read your paper and saw myself, and tears run down my face as I write this. The quote you provided from another father regarding this loss as being “world changing” is highly descriptive.

Thank you for your work.

At first, we were lost for words, we were so deeply touched by this response to our article, so moved by what he wrote about the title and about his wife listening to the song. We were also grateful for the feedback indicating that Norwegian parents’ experiences are recognisable in other parts of the world. Of course, we went on to respond to him personally and we also drew his attention to a further article that at the time was accepted in *OMEGA—Journal of Death and Dying*, the one that triggered further correspondence between us, the one which is the subject of this commentary. To step back a little and explain: These two publications are reports on a large-scale, still-ongoing research project that began in 2017 (The END-project), focusing on drug-death bereavement and recovery from the perspective of bereaved family members, close friends and community helpers. This project is close to the hearts of all three of us, as it also is to the extended team of END researchers involved in working on different aspects, all relating to DRD. We are keen to disseminate our findings, for both scientific and applied purposes: To add to knowledge and increase understanding of DRD bereavement, on the one hand, and to contribute to support for DRD bereaved persons and those who care for them professionally and personally, on the other hand. So when we received a second email from Kelly Thomas about the *OMEGA—Journal of Death and Dying* article, we were moved to suggest its publication as a commentary piece in *OMEGA—Journal of Death and Dying*: Kelly Thomas’s words vividly illustrate the points we covered in our qualitative analysis. Of course we ourselves included illustrations from the interviews with the participants, in our articles. But here we have an entirely different narrative, namely, the account of one particular DRD bereaved father actually reflecting on the categories—scientific constructions as they are—of feelings and behaviors and attitudes that we identified in our qualitative analysis. We learn from Kelly Thomas how our analysis “spoke” to someone who has himself had to endure the loss of a loved child to DRD. His words speak for themselves.

## Kelly Thomas’s Commentary on Titlestad et al. (2020)

Since I have now benefited from two of your papers, I was moved to provide a brief summary of my experience relative to that described in your paper on the off-chance that this might offer any insights to you. I was struck by the similarity between the experiences reported by DRD bereaved parents in Norway and my own. The separation of an ocean and a culture, both vast, seems to do little to separate my experience from theirs. Hence I offer the following, organized as you did in the Results section of this paper. As background, we lost my daughter, Cara, 3 years ago (on 5/21/17) at age 21. She had suffered from addiction in one form or another since around age 15, with the addiction increasing in intensity after age 18. Cara had mental health challenges from a young age (pronounced insecurity, impulsive behaviour, etc.), all of which were aggravated by a sexual assault at age 10, which she kept from her mother and me until she was in treatment for addiction. Cara’s death was violent and was not a suicide.

### Processing Grief Emotions

#### Ruminating About Guilt

Repetitive and recurrent thinking about my guilt has been a constant. The intensity of these feelings has declined with time, albeit not as a monotonically decreasing function. It has helped, somewhat, that others close to me have repeatedly assured me that I was a good parent and that this was not my fault. It also helped, significantly, that we joined a group of bereaved parents that meets weekly; this allows me to see that this is a common reaction, not unique to me. At the core, however, I am her father and was responsible for her safety and well-being, and I was clearly not successful; I expect this feeling to stay with me to some extent as long as I live.

#### Reflections on Blaming Others

This was a very intense initial feeling for me. I blamed the people she was involved with as an addict, and particularly those involved with her death. These feelings declined pretty quickly for me (i.e., within 6 months or so), as I recognized they are all addicts or involved in the drug trade, and hence crippled themselves. I also felt a lot of resentment towards the “system” here in the US, which is not optimal in terms of getting real help for those suffering from drug addiction; insurance companies exist to generate profits, not to actually help those they ensure, and government programs are woefully inadequate. Getting Cara adequate help was very much a constant uphill battle. However, at the end of the day, we were able to get her quite a bit of support, and it still did not save her. A resentment that is with me fairly strongly still is towards the police and legal authorities in the area where Cara was killed. To these people, my daughter was just another dead addict, and they have a new pile of dead addicts every week; I cannot move past my disgust at their callousness.

#### Adaption to External Triggers

I learned fairly quickly what my external triggers were (specific places, specific types of music, etc.) and learned to expose myself to these in small doses. The one I could not avoid was Cara’s son, my grandson, who my wife and I are now raising. He was 2 when Cara passed away, but we had taken over raising him (required a court order) shortly before he turned 1 since her lifestyle was not safe for him. I see Cara in him daily, so that is a “constant trigger”. I also force myself to talk with him about Cara often, and he has grown used to seeing my tears when we do, although my emotions are less intense now and I am better able to have these discussions with him without breaking down.

### Proactive Coping

#### Cognitive Strategies

In this area, I try to be aware that I need to “set aside” time to think and talk about my daughter frequently to avoid “bottling it up” and having my emotions come out in destructive ways. I think about this as “titrating my grief”. It helps that, as noted above, I belong to a group of bereaved parents that meets weekly, so I am forced to confront this deeply at least once a week. We also had my grandson in a Children’s Bereavement Center program for about 2 years, which met every other week, and the “grandparents” in the group met separately, which helped as well.

#### Communication Strategies

I avoid talking about my loss with anyone except other bereaved parents, as I don’t believe someone who has not gone through this could understand. I have talked a little with friends, but I know they are incapable of actually understanding. I am painfully aware I was unequipped to discuss this topic before suffering my own loss. It helps that I belong to a group of bereaved parents that meets weekly, and that is my primary outlet for talking about this; the dads from this group get together for dinner once a month, and I can call/text other dad’s when needed. The bereavement program I had my grandson in provided a secondary outlet. I joined a Facebook group for bereaved fathers, and that provided an additional outlet, albeit an on-line group is a much less effective outlet than a local group where you meet face-to-face. As noted above, I talk often about Cara with her son. I also talk with my wife, my other daughter and her husband, and my son and his wife.

#### Craving Knowledge

I knew this would not help me, and so did very little of it. That which I did was to build a set of documentation which we used to try to force the legal authorities to take action, which turned out to be a waste of effort.

#### Back to Day-to-Day Activities

Having my grandson to raise provided a lot of structure to my life, which was extremely helpful. I returned to work after 2 weeks, mainly because I felt that I needed to in order to avoid thinking of my daughter & her death 24 hours a day. My employer told me to take all the time I need and suggested 2 weeks might be too soon. They also took a very hands-off approach upon my return, allowing me to “come up to speed” over a period of a few months. They were extremely supportive. I work for a relatively small engineering company (150 staff) and we are very flexible in terms of supporting our staff, and I fully recognize how lucky I am that this is the case.

### Giving and Receiving Support and Assistance

#### To be Needed by Others

Having my grandson to raise was vitally important to my recovery. My other children were already married and did not “need me”, and I am very much aware that it would have been much harder for me without my grandson. I was also helped by the knowledge that my wife needed me not to completely and totally fall apart. It also helped that I knew, to some extent, I was needed at my workplace. Once I got past the first year, I also came to recognize that others new to the bereaved parents group “needed” me as an example that they could also deal with this and to provide practical advice on how they might do so.

#### Social Network Support

I did not, and do not, really look to my normal social network (i.e., family, friends and/or colleagues) for support since, as indicated earlier, I do not really believe anyone who has not gone through this can understand. I have relied much more heavily on grief support groups. One of the pastors at my church has also been very supportive and helpful; she loved my daughter and cared for her deeply, and that connection provides a bridge that she is able to use to reach me.

#### Professional Assistance

In the US, you are generally left to seek out support on your own. As discussed above, the bereaved parents’ group has been very important to me; it is sponsored by a local funeral home (i.e., as a way of giving back to the community). I am very lucky to live in San Antonio, where there is both a group for bereaved parents as well as a Children’s Bereavement Center; there are many places in the US that do not have these resources available. The Facebook group (bereaved fathers) is helpful, but nothing like an in-person group. My wife and I also went jointly to counselling for a few months, and then I went individually for a year, and both were very helpful in that they forced me to confront my loss and my feelings about it. My GP helped as well in that I was unable to sleep very much (due to dreams), and he prescribed a medication that allowed me to do so (zolpidem), which I still take; he has provided my name to his other patients who have suffered the loss of a child as a resource to help them.

#### Peer Support

I have noted the importance of peer groups above. A face-to-face peer group for fathers who have experienced DRD would be ideal. However, the face-to-face bereaved parents’ group, albeit their losses came in many forms, is still very important and helpful to me; they are able to directly relate to my loss despite the different circumstances under which they lost a child.

Last, the oscillation between different modes/approaches was very much my experience the first year. In the second year, and now more so in the third, I have reached more of a steady-state. As discussed earlier, particularly early on, I found the need to titrate my grief to prevent it from overwhelming me. Late in the second year, I found that the waves had decreased in both amplitude and frequency, albeit a rogue wave does arrive now and then, and I suppose this will be the case from here on out. Hence, there is less need now for me to try to “control” my grief. I am sure talking through my feelings with the bereaved parents’ groups, and with counsellors, has helped me reach the point. As discussed earlier, my role in raising my grandson, and talking about my daughter with him, has also been critical in dealing with my grief.

I hope that this summary of my experience provides you with some additional insights, or at least increased confidence in the conclusions you have drawn to date. Thank you again for your work, and please accept my best wishes and encouragement for your continued endeavours in this area.

## Authors’ Comments

We were struck by this eloquent, open, deeply-insightful, heart-rending—but also heart-warming—account portrayed by Kelly Thomas of his reactions in general and grief in particular. We have exchanged many thoughts between the three of us, thinking about what he shared with us, and about how to respond here. There is so much we would like to add! However, on reflection, we agreed that detailed comments, for example, on comparability with our research participants’ experience in the different cultural settings, would only detract from the impact of Kelly Thomas’s personal narrative. So here we limit our comments to a couple of take-home messages and general remarks which were prompted by his insights.

As Kelly Thomas himself points out, DRD bereavement experiences cross borders: He mentioned how he found that our qualitative descriptions with Norwegian parents mirrored his own experience within the very-different U.S. culture. It is encouraging to think that our research has international relevance/significance. The extent to which this is the case is something for future investigation, a topic on which research teams across different countries could usefully collaborate.

Further research on this topic in general is called for: In these times of COVID-19, when understandably, so much attention is being paid to bereavement following this type of loss, it is important to retain a balance, to draw attention back to other types of losses, such as DRD bereavement, to highlight the occurrence of such individual tragedies too.

A more specific suggestion for future research emerges: It is important to recognize high risk sub-groups. The challenges faced by some young persons like Kelly Thomas’s daughter are familiar to us from the Norwegian interviews. For example, their difficulties in adapting to the expectations of the school system became evident. As in Kelly Thomas’s daughter’s case, these are sometimes associated with impulsive behavior. Parents in our own study described, for example, how sexual assault had afflicted the daughter who had died of DRD, the experience having affected her ongoing life and as here, with the assault unknown to the parents until after the daughter had started to use narcotics.

It has become amply evident from Kelly Thomas’s account of the death of his daughter, Cara, that losing a loved one to DRD leaves behind an excruciating aftermath. We are deeply grateful to him for his willingness to share his personal experiences not only with us, the research team, but also with readers of *OMEGA—Journal of Death and Dying* across the world. We feel he has promoted our endeavor to draw attention to DRD bereavement.

## References

[bibr1-0030222820946550] Titlestad K. B. Mellingen S. Stroebe M. Dyregrov K. (2020). Sounds of silence. The “special grief” of drug-death bereaved parents: A qualitative study. *Addiction Research & Theory* Advance online publication. 10.1080/16066359.2020.1751827

[bibr2-0030222820946550] Titlestad K. B. Stroebe M. Dyregrov K. (2020). How do drug-death bereaved parents adjust to life without the deceased? A qualitative study. *OMEGA—Journal of Death and Dying*, *82*(1), 141–164. 10.1177/0030222820923168 PMC7418270 32389093

